# Plasma soluble P-selectin correlates with triglycerides and nitrite in overweight/obese patients with schizophrenia

**DOI:** 10.1515/pteridines-2020-0012

**Published:** 2020-05-23

**Authors:** Katelynn A. Bourassa, Teodor T. Postolache, Aline Dagdag, Dietmar Fuchs, Olaoluwa O. Okusaga

**Affiliations:** Michael E. DeBakey VA Medical Center, Houston, TX, USA; Mood and Anxiety Program, Department of Psychiatry, University of Maryland School of Medicine, Baltimore, MD, USA, Veterans Health Administration, Rocky Mountain Mental Illness Research Education and Clinical Center (MIRECC), Denver Veterans Affairs Medical Center (VAMC), Aurora, CO, USA, Military and Veteran Microbiome Consortium for Research and Education (MVM-CoRE), Aurora, CO, USA, VISN 5 Capitol Health Care Network Mental Illness Research Education and Clinical Center (MIRECC), Baltimore, MD, USA; Mood and Anxiety Program, Department of Psychiatry, University of Maryland School of Medicine, Baltimore, MD, USA; Division of Biological Chemistry, Biocenter Innsbruck Medical University, Innsbruck, Austria; Michael E. DeBakey VA Medical Center, Houston, TX, USA, Menninger Department of Psychiatry and Behavioral Sciences, Baylor College of Medicine, Houston, TX, USA

**Keywords:** schizophrenia, overweight/obesity, P-selectin, nitrite, atherosclerosis

## Abstract

**Background:**

Soluble P-selectin (sP-selectin) is associated with risk factors for cardiovascular disease (CVD) but this association has not been evaluated in patients with schizophrenia. This study primarily evaluated the association of sP-selectin with plasma lipids and nitrite (NO_2_−) respectively in overweight/obese adults with schizophrenia.

**Methods:**

One-hundred and six patients with schizophrenia (mean age 32.9 years; 71.60% male) were recruited from a psychiatric hospital. Participants completed a structured interview and provided a fasting blood sample. Body mass index (BMI) was used to divide the sample into normal weight and overweight/obese groups. Pearson’s and partial correlation coefficients (controlling for age, sex, race, education, and inflammation) were calculated to examine the association of sP-selectin with plasma lipids, and NO_2_− in the overweight/obese patients (primary analysis), as well as in the normal weight patients and the total sample (exploratory analyses).

**Results:**

After controlling for potential confounders, sP-selectin positively correlated with triglycerides (*r* = 0.38, *p* = 0.01) and NO_2_− (*r* = 0.40, *p* < 0.01) in the overweight/obese group only.

**Conclusions:**

Future longitudinal studies should evaluate the utility of sP-selectin as a biomarker of CVD in overweight/obese adults with schizophrenia (for example, by relating sP-selectin to incidence of cardiovascular events).

## Introduction

Patients with schizophrenia have increased rates of cardiovascular disease (CVD)-related morbidity and mortality, resulting in approximately 11-20 years decreased lifespan and up to 2.5 greater odds of dying in comparison to adults without a psychiatric illness [1, 2]. Overweight/obesity is an important factor contributing to CVD in patients with schizophrenia [3]. Also, overweight/obesity in schizophrenia is considered to be in great part a consequence of certain antipsychotic medications [4] and is linked to a high rate of metabolic syndrome in this population [5].

Atherosclerosis, one of the main causes of CVD, is characterized by an initial stage of endothelial damage and activation [6]. After damage and activation, the endothelium expresses adhesion molecules, including P-selectin, which mediate leukocyte adhesion, rolling and migration into the subendothelial region [7]. P-selectin is an important factor for atherogenesis as highlighted by the fact that P-selectin knockout mice exhibit negligible atherosclerosis [8] while mice genetically modified to overproduce soluble P-selectin (sP-selectin) exhibit accelerated rates of atherosclerosis [9]. sP-selectin is derived from proteolytic cleavage of membrane-bound P-selectin or from alternative mRNA splicing [10]. Plasma sP-selectin has been found to be associated with waist circumference and other CVD risk factors (triglycerides, total cholesterol, smoking) in non-psychiatric samples [11]. Interestingly, elevated plasma sP-selectin has also been found in patients with schizophrenia [12].

In addition to sP-selectin expression, the initial stage of atherosclerosis is also characterized by reduced bioavailability of nitric oxide (NO) [13]. NO is synthesized in the body through the L-arginine-NO pathway which is mainly regulated by three isoforms of the enzyme nitric oxide synthase (NOS) namely endothelial NOS (eNOS), neuronal NOS (nNOS) and inducible NOS (iNOS); however, eNOS and nNOS are the primary regulators under physiological conditions [14]. The reduced bioavailability of NO during atherogenesis is related to its inactivation secondary to oxidative stress [15], a phenomenon that has also been associated with obesity in schizophrenia [16]. NO has a very short half-life therefore most investigators estimate NO level by measuring levels of nitrate (NO_3_−) or nitrite (NO_2_−), both of which are metabolites of NO [17]. Of note, polymorphism of the *NOS3* gene (codes for endothelial NOS, eNOS), specifically the T-786C SNP, is associated with risk of metabolic syndrome in schizophrenia [18].

We previously found that in overweight/obese patients with schizophrenia (but not in normal weight patients), peripheral inflammation (assessed by plasma high sensitivity C-reactive protein [hs-CRP]) and triglycerides (TG) were elevated, while high-density lipoprotein-cholesterol (HDL-C) level was low [19]. Since dyslipidemia and NO are both involved in the pathophysiology of atherosclerosis, we now aimed to evaluate the association of sP-selectin with plasma lipids and NO_2_− in the same overweight/obese patients with schizophrenia. We hypothesized that in overweight/obese patients with schizophrenia, sP-selectin would positively correlate with plasma total cholesterol (TC), TG, and low-density lipoprotein-cholesterol (LDL-C), but negatively correlate with HDL-C and NO_2_−. On an exploratory basis we also evaluated the association of sP-selectin with plasma lipids and NO_2_− in the total sample (i.e. normal weight and overweight/obese patients combined) and in normal weight patients alone.

## Methods

### Study sample

One-hundred and six participants were enrolled in this cross-sectional study. All participants had a diagnosis of schizophrenia and were receiving antipsychotic medication. To be included in the study, individuals were required to: (1) be between the ages of 18 and 60, (2) have a documented diagnosis of schizophrenia consistent with DSM-5 criteria, and (3) have a negative urine pregnancy test if female. Exclusion criteria included: (1) active suicidal and/or homicidal ideation, (2) a history of a primary cognitive disorder, (3) a current primary inflammatory condition and/or infectious disease, (4) receipt of corticosteroids or non-steroidal anti-inflammatory medication, (5) receipt of warfarin or other anticoagulant medication, and (6) current use of psychostimulant drugs, evidenced by a positive urine drug screen. Participants were recruited from the inpatient unit of a university-affiliated psychiatric hospital after approval by the Institutional Review Board.

Information on sex, age, race, and level of education were obtained from each participant. The Mini International Neuropsychiatric Interview (a structured diagnostic interview) [20] was used to confirm the diagnosis of schizophrenia. Weight classification was approximated by calculating BMI. Specifically, BMI was defined as weight (in kilograms) divided by the square of height (in meters). Participant’s height was measured with a stadiometer and weight with an adult beam balance scale. We categorized participants with BMI ≥ 25 kg/m^2^ as overweight/obese and those with BMI ranging from 18.5 kg/m^2^ to 25 kg/m^2^ as normal weight.

#### Ethical approval:

The research related to human use has complied with all the relevant national regulations, institutional policies and in accordance with the tenets of the Helsinki Declaration, and has been approved by the authors’ institutional review board or equivalent committee.

#### Informed consent:

Informed consent has been obtained from all individuals included in this study.

### Measurement of plasma sP-selectin, lipids, NO_2_−, and hs-CRP.

Fasting venous blood samples were collected (between 6am and 7am) in EDTA-containing tubes, and plasma was isolated and stored at −80 degrees Celsius until analyzed. sP-selectin was measured using ELISA (Enzyme-linked immunosorbent assay) kits according to the manufacturer’s (RayBiotech, Inc. Georgia, USA) instructions. Samples were diluted 1:50 after which optical density values for the samples were compared to standard curves ranging from 0-30ng/ml (Assay sensitivity was 20 pg/ml). Plasma TC, TG, LDL-C, and HDL-C were measured by Quest Diagnostics (Secaucus, NJ) using spectrophotometry. hs-CRP was measured using an ELISA kit (MyBioSource, MBS703598); in preparation for analysis, samples were diluted 1:1000 and the procedures were consistent with the manufacturer’s instructions. Plasma nitrite was measured following the protocol outlined by Giustarini and colleagues (2004) [21] using a modified Griess-Ilosvay diazotization reaction assay (Merk KGaA, Darmstadt, Germany). The protocol included the reaction of sulfanilamide with NO_2_− present in samples acidified with phosphoric acid, forming a diazonium salt. N-(1-naphthyl) ethylenediamine dihydrochloride is then coupled with the diazonium salt forming an azo dye which was subsequently analyzed at 562 nm in a spectrophotometer (KC4 reader, Bio-Tek Instruments, Inc., Winooski, VT, USA). The measurement of plasma NO_2_− was carried out at the Division of Biological Chemistry, Biocenter, Medical University of Innsbruck, Austria.

### Statistical Analyses

Variables were first examined for normality. sP-selectin, hs-CRP, and NO_2_− were skewed to the right and a log transformation was performed on each variable. Means ± standard deviation or percentages (as appropriate) are presented for descriptive variables. For sP-selectin, hs-CRP, and NO_2_−, we have presented geometric means obtained by exponentiating the mean log-transformed values. To examine the primary hypothesis that plasma sP-selectin is associated with plasma lipids and NO_2_− in overweight/obese patients, Pearson’s correlations were calculated with partial correlations to control for potential confounders (i.e., sex, age, race, education and inflammation [i.e. hs-CRP]). On an exploratory basis, the correlations (Pearson’s and partial) of sP-selectin with plasma lipids and NO_2_− were examined in the normal weight patients and the entire sample (i.e., overweight/obese and normal weight combined). Exploratory analyses also included t-test to compare sP-selectin and NO_2_− between normal weight and overweight/obese patients. All tests were two-tailed. To control for type I error, Bonferroni correction was applied in the primary analyses (i.e. in overweight/obese patients only), while alpha was set at 0.01 for exploratory analyses. All analyses were performed in IBM SPSS, Version 24 (IBM Corp., Armonk, NY, USA).

## Results

### Sample characteristics

The overall sample was approximately 33 years of age, on average (*M* = 32.9, *SD* = 12.3), predominantly male (*n* = 76; 71.6%), and a slight majority were African American (*n* = 55; 51.9%). Most participants had a high school diploma or equivalent (*n* = 38; 35.9%) at the time of the study. A slight majority of the sample had a BMI in the overweight/obese range (*n* = 54, 53.5%). Differences in plasma lipids between overweight/obese and normal weight patients are reported elsewhere [19]. See Table 1 for a depiction of sample characteristics.

### Association of sP-selectin with lipids and NO_2_− in overweight/obese patients

Results from unadjusted analyses indicated that sP-selectin was positively correlated with TG (*r* = 0.40, *p* < 0.01) and NO_2_− (*r* = 0.38, *p* < 0.01), but had no significant correlation with TC, LDL-C, or HDL-C. The positive correlations between sP-selectin and TG and NO_2_− respectively, remained significant after controlling for potential confounders (*r* = 0.38, *p* = 0.01 and *r* = 0.40, *p* < 0.01 respectively). See Table 2.

### Association of sP-selectin with lipids and NO_2_− in the normal weight participants and in the total sample

sP-selectin was not associated with plasma lipids or NO_2_− in either normal weight patients or in the total sample.

### Comparison of sP-selectin and NO_2_− in normal weight and overweight/obese participants

sP-selectin and NO_2_− did not differ between the normal weight and overweight/obese patients (43.35 ± 0.18 ng/ml vs. 38.02 ± 0.18 ng/ml, p=0.13 and; 21.44 ± 0.27 μmol/L vs. 24.14 ± 0.32 μmol/L, p=0.39 respectively) in this sample.

## Discussion

We found, for the first time (to our knowledge), a positive correlation between sP-selectin and both TG and NO_2_− respectively in overweight/obese patients with schizophrenia but not in normal weight patients; however, we did not observe any association between sP-selectin and TC, LDL-C, or HDL-C. Our finding of a positive relationship between sP-selectin and TG in overweight/obese adults with schizophrenia is consistent with the limited literature in non-psychiatric samples. For example, Belinski et al [22] found a positive correlation between sP-selectin and both TG and LDL-C but a negative correlation with HDL-C in the Multi-Ethnic Study of Atherosclerosis.

Although we could not find any previous study involving patients with schizophrenia, research involving animals [23, 24] and non-psychiatric human participants [25–27] have related sP-selectin with NO_2_− but they evaluated sP-selectin expression using other techniques (e.g. radiolabeled monoclonal antibody technique, Western blot and flow cytometry) [24, 26, 27] rather than plasma levels as in our study. In the previous studies, there was a consistent finding of a negative correlation between sP-selectin and NO_2_− (as well as nitrate [NO_3_−]). For example, exogenously administered NO_2_− (e.g. dietary NO_2_−) attenuated P-selectin [28–30] while inhibition of NO (and therefore NO_3_− and NO_2_−) biosynthesis increased P-selectin expression [26, 27]. Contrary to previous studies and our hypothesis, we found a positive correlation between sP-selectin and NO_2_− in the subsample of overweight/obese patients with schizophrenia after controlling for potential confounding factors. A plausible explanation (though speculative) for our finding is that NO synthesis increases in parallel with sP-selectin in overweight/obese patients as an attempt to counteract the procoagulant and atherogenic effects of sP-selectin and the atherogenic effect of TG. It is also possible that the expression of iNOS is increased in the overweight/obese patients since iNOS expression is increased in obesity-induced inflammatory states [31] and as described earlier, levels of inflammation were higher in the overweight/obese patients [19]. It is, however, important to note that there was no difference in NO_2_− levels between overweight/obese patients and normal-weight patients in this sample.

Regarding the association of sP-selectin with TG in the overweight/obese patients, it is plausible to speculate that it could be an indication of ensuing pathology in the vasculature related to the combination of elevated inflammation and hypertriglyceridemia in the overweight/obese patients [19]. This inference is consistent with the report of a systemic inflammatory environment inducing a phenotypic switch to a proatherogenic endothelium which leads to enhanced expression of P-selectin [32, 33]. The potential importance of our observed association is also highlighted by the fact that recent data suggest that when standardized for their respective effects on apolipoprotein B (ApoB), TG might confer similar risk of CVD as LDL-C, an established CVD risk factor [34, 35]. However, we did not observe higher levels of sP-selectin in the overweight/obese patients relative to normal weight patients, a finding that is inconsistent with the preceding arguments and requires further exploration.

Several limitations should be considered when interpreting the findings of the current study. First, the average BMI in our study population was at the lower end of the overweight category (i.e., 26.99±7.03 kg/m^2^), and it is possible that the relationship among sP-selectin and plasma TG and NO_2_− may be stronger in a more obese population. Additionally, average plasma lipid levels were within the normal range for the total sample, which may have resulted in an attenuated relationship with sP-selectin. Second, given the cross-sectional study design, causal relationships among sP-selectin, NO_2_−, TG, overweight/ obesity, cannot be established and the findings of this study may not generalize to other populations. Third, data on psychotropic medication type and dosage, important potential confounders, were not available for this study. As the affinity for weight gain differs among neuroleptics [36], we were unable to evaluate whether neuroleptics may differentially impact the relationship among sP-selectin, NO_2_−, and TG in adults with schizophrenia. Fourth, dietary sources contribute considerably to plasma NO_3_− and NO_2_− [37, 38] but since we did not collect information on dietary intake, the possibility that our results are related to diet, cannot be ruled out. Finally, we did not collect data regarding additional CVD risk factors (e.g., smoking status, metabolic syndrome, diabetes). We were unable to determine whether the presence of these factors confounded, mediated, or moderated the observed associations among sP-selectin, TG, and NO_2_− as suggested by prior research reporting sP-selectin elevation in the aforementioned conditions [39, 40].

### Future directions

The utility of sP-selectin as a potential biomarker of cardiovascular health in overweight/obese patients with schizophrenia should be evaluated in future prospective cohort studies by serial measurements of sP-selectin, lipids, NO_2_−, and relating them to the incidence of cardiovascular events (e.g. myocardial infarction and stroke). Other studies should also evaluate the efficacy of dietary nitrite and nitrate supplementation in overweight/ obese patients with schizophrenia since dietary nitrite and nitrate have been shown to increase bioavailability of NO and decrease triglyceride levels in a non-psychiatric sample [41]. Furthermore, in experimental models, dietary nitrite and nitrate supplementation attenuate the metabolic abnormalities (obesity, hyperlipidemia, glucose intolerance, inflammation and oxidative stress) induced by high-fat diet in mice [42]. The effect of weight loss interventions on sP-selectin and its association with lipids and NO_2_−, might be also evaluated in future studies since in non-psychiatric samples obese individuals who lost 10% (or more) of their body weight in six months exhibited reductions in endothelial dysfunction and platelet activation as evidenced by platelet aggregation studies and reduced sP-selectin levels [43].

In addition, longitudinal studies of medication naive patients are necessary to take into account the metabolic effects of neuroleptics [44] as well as their potential effect on vascular dysfunction [45]. Finally, collecting larger samples with greater variability in BMI status would allow for a more in-depth examination of the relationships among sP-selectin, plasma lipids, and NO in adults with schizophrenia who have BMIs in the normal, overweight, and obese ranges.

## Figures and Tables

**Table 1:** Sociodemographic and metabolic characteristics of the sample (N = 106).

Variable	Value
Sex (Male; *n* [%])	76 (71.60%)
Mean Age ±SD	32.90 ±12.28
Race *n* (%)	
Caucasian	33 (31.10%)
African American	55 (51.90%)
Hispanic	16 (15.10%)
Asian	2 (1.90%)
Education *n* (%)	
No Degree	34 (32.10%)
High School/Equivalent	38 (35.90%)
Some College	25 (23.60%)
>2 Years College	9 (8.40%)
Mean BMI±SD	26.99±7.03
BMI Category *n* (%)	
Normal	47 (46.50%)
Overweight/Obese	54 (53.50%)
Geometric Mean sP-selectin (ng/ml) ±SD	40.08±0.18*
Geometric Mean hs-CRP (mg/L) ±SD	1.05±0.62*
Mean Total Cholesterol (mg/dL) ±SD	157.53±32.05
Mean TG (mg/dL) ± SD	106.59 ± 56.89
Mean LDL-C (mg/dL) ±SD	91.46±29.89
Mean HDL-C (mg/dL) ±SD	44.75±14.40
Geometric Mean Plasma Nitrite μmol/L) ±SD	22.21±0.29*

* *Note.* = Geometric mean of log transformed variable; BMI = Body Mass Index; HDL-C = High Density Lipoprotein Cholesterol; hs-CRP = High Sensitivity C-Reactive Protein; LDL-C = Low Density Lipoprotein Cholesterol; sP-selectin = Soluble P-Selectin; TG = Triglycerides; 5 participants are missing BMI data.

**Table 2:** Correlations among sP-selectin, plasma lipids, and plasma nitrite in participants with BMI in the overweight/obese range (n = 54) before and after adjustment for sex, age, race, education, and hs-CRP.

	Pearson’s *r* sP-selectin (unadjusted)	*p* value	Pearson’s *r* sP-selectin (adjusted)	*p* value
TC	0.13	0.39	0.12	0.44
TG	0.40	< 0.01	0.38	0.01
LDL-C	0.08	0.60	0.07	0.67
HDL-C	−0.31	0.03	−0.31	0.04
Plasma Nitrite	0.38	< 0.01	0.40	< 0.01

HDL-C = High Density Lipoprotein Cholesterol; LDL-C = Low Density Lipoprotein Cholesterol; *r* = correlation coefficient; TC = Total Cholesterol; TG = Triglycerides; *n* = 50 provided data for TC and HDL-C; *n* = 49 provided data for TG and LDL-C, *n* = 49 provided data for plasma nitrite.
